# Markers for Ongoing or Previous Hepatitis E Virus Infection Are as Common in Wild Ungulates as in Humans in Sweden

**DOI:** 10.3390/v8090259

**Published:** 2016-09-19

**Authors:** Anette Roth, Jay Lin, Lars Magnius, Marie Karlsson, Sándór Belák, Frederik Widén, Heléne Norder

**Affiliations:** 1 Department of Infectious Diseases, Institute of Biomedicine, University of Gothenburg, 413 46 Gothenburg, Sweden; anette.roth@microbio.gu.se (A.R.); marie.karlsson.2@gu.se (M.K.); 2 Department of Virology, Microbiology, National Veterinary Institute, 756 51 Uppsala, Sweden; jay_lin79@yahoo.se (J.L.); frederik.widen@sva.se (F.W.); 3 Department of Biomedical Science and Veterinary Public Health, Swedish University of Agricultural Sciences (SLU), 756 51 Uppsala, Sweden; sandor@belak.se; 4 The OIE (World Organisation for Animal Health) Collaborating Centre for the Biotechnology-Based Diagnosis of Infectious Diseases in Veterinary Medicine, 756 51 Uppsala, Sweden; 5 Ulf Lundahl Foundation, 116 21 Stockholm, Sweden; lars.magnius@gmail.com

**Keywords:** hepatitis E virus, zoonosis, moose, wild boar, deer, phylogenetic analysis, wild animals, Sweden

## Abstract

Hepatitis E virus (HEV) is a human pathogen with zoonotic spread, infecting both domestic and wild animals. About 17% of the Swedish population is immune to HEV, but few cases are reported annually, indicating that most infections are subclinical. However, clinical hepatitis E may also be overlooked. For identified cases, the source of infection is mostly unknown. In order to identify whether HEV may be spread from wild game, the prevalence of markers for past and/or ongoing infection was investigated in sera and stool samples collected from 260 hunted Swedish wild ungulates. HEV markers were found in 43 (17%) of the animals. The most commonly infected animal was moose (*Alces alces*) with 19 out of 69 animals (28%) showing HEV markers, followed by wild boar (*Sus scrofa*) with 21 out of 139 animals (15%), roe deer (*Capreolus capreolus*) with 2 out of 30 animals, red deer (*Cervus elaphus*) with 1 out of 15 animals, and fallow deer (*Dama dama*) 0 out of 7 animals. Partial open reading frame 1 (ORF1) of the viral genomes from the animals were sequenced and compared with those from 14 endemic human cases. Phylogenetic analysis revealed that three humans were infected with HEV strains similar to those from wild boar. These results indicate that wild animals may be a source of transmission to humans and could be an unrecognized public health concern.

## 1. Introduction

Hepatitis E virus (HEV) is a small non-enveloped virus with a virion size of approximately 27–34 nm in diameter [1]. It has a positive sense, single-stranded RNA of 6.6–7.3 kb. The genome is organized into three open reading frames (ORF1–3). ORF1 and 3 encode non-structural proteins, and ORF2 encodes the viral capsid protein [2]. 

HEV belongs to the *Hepeviridae* family, which has recently been split into two genera, *Orthohepevirus* and *Piscihepevirus* [3]. The only *Piscihepevirus* so far known, the cutthroat trout hepevirus, forms the species *Piscihepevirus* A [4]. *Orthohepevirus* is classified into four species designated *Orthohepeviruses* A–D. The strains infecting humans belong to *Orthohepevirus* A, which is formed by seven genotypes, HEV1–HEV7 [3]. HEV1 and HEV2 infect only humans, while HEV3 and HEV4 also infect several mammalian species such as domestic pigs, wild boar, rabbit, deer, rat, and mongoose [4]. HEV3 strains infecting swine, rabbits, and humans have been described from the Americas, Europe, Asia, Oceania, and recently Africa [5,6,7]. Strains belonging to HEV4 have mainly been found in Asia, but recently also in Europe [8,9,10,11]. Domesticated pigs and wild boars are believed to be an important source of zoonotic transmission of HEV3 and HEV4 and responsible for sporadic human hepatitis E cases worldwide [12,13]. HEV5 and HEV6 have been identified from infected Japanese wild boar, and HEV7 from infected camels [14,15,16]. There is an additional divergent HEV type infecting moose [17]. It is not known whether these latter genotypes and variants also infect humans. 

HEV is considered to be one of the major causes of acute viral hepatitis worldwide. The World Health Organization (WHO) estimates that there are globally around 20 million cases annually, resulting in approximately 56,600 deaths. In developing countries, HEV1 and HEV2 may cause serious outbreaks, mainly through contaminated water. The mortality is up to 30% in HEV1-infected pregnant females in the third trimester [18,19]. In Europe, the USA, and Japan, HEV3 is commonly spread through the fecal-oral route [4,20,21,22], but may also be blood-borne [22,23,24]. The infection is usually self-limited in otherwise healthy persons, but may cause liver failure or chronic infection in patients with underlying liver disease or immunodeficiency [25]. It may also cause neurological manifestations [26]. The seroprevalence of HEV in countries where HEV3 is endemic varies from 0.3% to 30% [6,24,27,28,29,30]. Despite a rather high seroprevalence of HEV in Sweden (17%) [29], less than 30 cases of hepatitis E are reported annually to the public health authorities [31]. This may be due to most infections being subclinical, but also due to the treating physician’s unawareness of endemic hepatitis E. When an endemic case is identified, the route of infection almost always remains unknown. Since it is known that HEV infection is common among swine and wild boar in Europe, Swedish ungulates were investigated for this virus. The two most common wild ungulates in Sweden are wild boar and moose.

The wild boar (*Sus* *scrofa*) is a withdrawn nocturnal animal that lives in a matriarchate. Wild boars were once common but hunted to extinction in the 18th century. After 200 years of absence, they were reintroduced and have now established a wild population that has increased rapidly in the southeastern part of Sweden. According to the Swedish Hunting Association, the wild boar population was estimated to be 40,000 in 2005–2006, and in June–July of 2010–2011 it had increased to 150,000 [32]. During the 2012–2013 season, 97,300 wild boar were hunted and killed in Sweden [33].

The moose (*Alces* *alces*) is the largest member of the deer family. It inhabits boreal and mixed deciduous forests and is found all over Sweden except for Gotland. Its population increased until the beginning of the 18th century, but moose were then hunted almost to extinction. Around 1830, it was estimated that only 200 moose were left. Today, the summer population of moose in Sweden is estimated to be 300,000–400,000. Each autumn, approximately 100,000 moose are hunted and killed [33].

This study was conducted to examine whether wildlife may represent a source of human HEV infections. The prevalence of markers of past and ongoing HEV infection in wild ungulates was investigated, and a phylogenetic analysis was carried out to compare the viral genomes of strains isolated from wild animals with those isolated from humans.

## 2. Materials and Methods 

### 2.1. Samples

A total of 490 samples from 260 animals were analyzed in this study. There were 250 serum samples and 240 stool samples from 139 wild boar (*Sus scrofa*), 69 moose (*Alces alces*), 30 roe deer (*Capreolus capreolus*), 15 red deer (*Cervus elaphus*), and seven fallow deer (*Dama dama*). The results from analysing the samples from 57 of the 69 moose have been described previously [34]. The samples were collected between 2012 and 2015 by 13 hunting teams in eight different provinces of Sweden. After shooting an animal, the hunter took stool and blood samples and sent them to the Department of Clinical Microbiology-Virology at Sahlgrenska University Hospital (Gothenburg, Sweden) together with a form filled in with the date of the kill, animal species, and an estimate of the age of the animal. All samples were kept at −20 °C until analysis. The animals were sampled in accordance with European legislations on animal research (approval numbers C194/7 and C124/11 issued by the ethical committee on animal research in Uppsala, Sweden). HEV strains were also analyzed in sera from 14 human cases of endemic hepatitis E in Sweden with unknown source of infection. The study was approved by the Regional Ethical Review Board in Gothenburg, with the registration number 737-12. 

### 2.2. Total HEV Antibodies (Anti-HEV) Detection

All serum samples were analysed for total anti-HEV antibodies (anti-HEV immunoglobulin (Ig) G and IgM simultaneously) by using HEV Ab EIA kit (Axiom Diagnostics, Worms, Germany) according to the manufacturer’s instructions. The sensitivity and specificity of this assay for the detection of human anti-HEV have been evaluated [29], and it has previously been used for the detection of non-human anti-HEV [35]. The microplates were read in an Infinite F50 microplate reader (TECAN, Grödig, Germany) at 450 nm using 650 nm as the reference wavelength.

### 2.3. HEV RNA Detection

#### 2.3.1. Nucleic Acid Extraction

About 1 g of stool sample was homogenized in tubes containing glass beads in phosphate-buffered saline (PBS; pH 7.4). The suspensions were centrifuged at 2700× *g* for 10 min. 

Two hundred fifty microliters of serum or supernatant from the stool suspensions were lysed and nucleic acids were extracted by using NucliSens^®^ easyMag^®^ instrument and reagents (Biomerieux, Marcy l’Etoile, France) and eluted in 110 µL of extraction buffer according to the manufacturers’ protocol.

#### 2.3.2. Reverse Transcription Real-Time PCR (RT-qPCR) Assay

The blood and stool samples were analyzed for HEV RNA in a one-step RT-qPCR assay targeting the ORF2/3 region of HEV1–HEV4 [17,34]. Each 50-µL reaction mix contained 20 µL of extracted RNA, 25 µL of 2× universal master mix and 1 µL Superscript^®^ III Platinum One-Step Quantitative RT-PCR System with ROX (Invitrogen, Carlsbad, CA, USA), 40 U of RNaseOUT^™^ (Invitrogen), and 0.2 µM of each primer and probe (Table 1).

Extracted nucleic acids from blood and fecal materials from moose, roe deer, red deer, and fallow deer were also analyzed for HEV RNA with a RT-qPCR assay, targeting the ORF2/3 region of moose HEV [34]. Each 50-µL reaction mix contained 10 µL of the extracted nucleic acids, 6 µL of RNase-free H_2_O (Sigma, Saint Louis, MO, USA), 25 µL of 2× universal master mix and 1 µL of Superscript^®^ III Platinum One-Step Quantitative RT-PCR System with ROX (Invitrogen), 40 U of RNaseOUT^TM^ (Invitrogen), 0.6 µM of each primer, and 0.2 µM of probe (Table 1).

The cycling conditions for both RT-qPCR assays were as follows: 50 °C for 30 min, 95 °C for 10 min, followed by two-step cycling 45 times at 95 °C for 15 s and 60 °C for 60 s. All RT-qPCR reactions were carried out on a 96-well plate on an ABI 7300 Sequence Detection System (Applied Biosystems, Foster City, CA, USA).

#### 2.3.3. cDNA Synthesis

cDNA synthesis was carried out in a 20-µL reaction mix containing 5 µL of extracted RNA, 200 U of SuperScript^®^ III (Invitrogen), 1× First Strand Buffer (Invitrogen), 5 mM of dithiothreitol (DTT, Invitrogen), 40 U of RNase OUT^TM^ (Invitrogen), 0.5 mM of deoxynucleoside triphosphate (dNTP) (Roche Diagnostics, Mannheim, Germany) and 4 µg of random hexamer primers (Roche Diagnostics).

#### 2.3.4. PCR Amplification for Sequencing

To amplify HEV3 strains for sequencing, a semi-nested PCR in ORF1 was performed in a 50-µL reaction mix with 5 µL of cDNA as template. The PCR mix contained 1× Taq buffer (Applied Biosystems), 2.25 mM of MgCl_2_ (Applied Biosystems), 0.2 mM of dNTP (Roche Diagnostics), 0.3 mM of each primer, and 1 U of Taq polymerase (Roche Diagnostics). Primers ISP4232-Pool 0-H and EAP4576-Pool 0-H were used for the first round PCR (Table S1). The PCR reaction was performed with initial denaturation at 94 °C for 3 min followed by 40 cycles with 94 °C for 20 s, 60 °C for 30 s, and 72 °C for 60 s. An aliquot of 5 µL of the first round product was used as a template in the second amplification round with primers ISP4232-Pool 0-H and IAP4561-Pool 0-G (Table S1) and 2.75 mM of MgCl_2_.

In order to detect moose-HEV, all blood and fecal samples from moose, roe deer, red deer, and fallow deer were also analyzed with a semi-nested PCR according to an earlier published protocol [34]. The 50-µL PCR mix contained 5 µL of cDNA, 1× Taq buffer (Applied Biosystems), 1 mM of MgCl_2_ (Applied Biosystems), 0.2 mM of dNTP (Roche), 0.3 mM of each primer, and 1 U of Taq polymerase (Roche Diagnostics). Primers ISP4232-A,B,E and EAP4576-E,F were used for the first round PCR (Table S1). The PCR reaction was performed with initial denaturation at 94 °C for 3 min followed by 40 cycles with 94 °C for 30 s, 56 °C for 30 s, and 72 °C for 65 s. An aliquot of 5 µL of the first round product was used as a template in a second amplification round with primers ISP4232-A,B,E and IAP4561-E,F,moose (Table S1) and 2.75 mM of MgCl_2_.

All amplified PCR products were purified and extracted with QIAquick PCR Purification Kit (Qiagen, Hilden, Germany).

#### 2.3.5. Controls Used in the Procedures

RNA stool and blood samples from swine or moose known to be positive for HEV were used as positive controls, and nuclease-free water was used as a negative control in all experiments (RNA extraction, cDNA synthesis, first and second round PCR amplification, and RT-qPCR assay).

#### 2.3.6. Sequencing

PCR products were sequenced using the same primers as for the nested PCR and the Big Dye Ready Reaction kit 3.1 (Applied Biosystems) according to the manufacturers’ instructions. Sequences were analyzed with the 3130xl Genetic Analyzer (Applied Biosystems). 

#### 2.3.7. Phylogenetic Analysis

The sequences obtained were analyzed with DNAStar SeqMan, package version 10.1.2 (DNA Star Inc., Madison, WI, USA). The sequences were aligned with the corresponding region of 347 sequences representing HEV3 and HEV4 obtained from GenBank. Phylogenetic analysis was carried out with the PHYLIP package version 3.65 [36]. Evolutionary distances were calculated using the F84 algorithm in the DNADIST program in the PHYLIP package with a transition-to-transversion ratio of 4.29. Phylogenetic trees were constructed using the unweight pair-group method using arithmetic averages (UPGMA) and the neighbor-joining method in the NEIGHBOR program of the PHYLIP package. The trees were visualized using the program Tree View, version 1.6.6. All sequences obtained in the study are deposited in GenBank with accession numbers KX757852–KX757878.

#### 2.3.8. Statistical Analysis

Statistical calculations were performed with Fisher’s test, using GraphPad Prism 6 (GraphPad software Inc., La Jolla, CA, USA). A *p*-value < 0.05 was considered significant.

## 3. Results

### 3.1. Anti-HEV Detection

When all available sera were analyzed for total anti-HEV antibodies, these were found in 23 out of 250 animals (9.2%). The highest frequency was found in moose (*Alces alces*) with 9 out of 66 (14%) of the animals having anti-HEV antibodies, followed by wild boar (*Sus scrofa*, 11 out of 134; 8.2%), the roe deer (*Capreolus capreolus*, two out of 29; 7%) and red deer *(Cervus elaphus*, one out of 14; 7%) (Table 2). None of the seven samples from fallow deer had anti-HEV antibodies that were detectable with the assay used. There was a tendency for animals older than one year to have detectable anti-HEV antibodies more often than the younger animals: 16 out of 129 (12%) versus 7 out of 117 (6%), respectively. This difference was, however, not significant (*p* = 0.1235; Fisher’s exact test). 

### 3.2. HEV RNA Detection 

When the two different RT-qPCRs developed in order to detect HEV1–4 and the moose HEV-RNA were used to analyse all the samples, HEV-RNA could only be identified in serum, fecal material, or both from wild boar and elk, and in sera from 14 human endemic cases. 

All samples were also analysed with two nested PCR, one developed for detecting HEV3 RNA and the other for detecting moose HEV RNA. 

HEV3 RNA could only be detected in samples from wild boar and from the 14 infected humans. Ten wild boar had HEV RNA in their serum or fecal materials, and one additional young animal had HEV RNA in both serum and feces (Table 2). 

When the PCR developed to identify the moose HEV-RNA was used to analyse all of the samples, it was only samples from moose that tested positive. Twelve moose had detectable HEV RNA, five of them in both stool and serum (Table 2). Three of these five moose were younger than one year. There was no significant difference with respect to the age of the animals (less than one year versus older than one year) and HEV RNA detection. 

None of the samples from deer had detectable HEV-RNA when tested with all the PCR assays described. 

### 3.3. HEV Markers (Anti-HEV and/or HEV RNA) and Geographical Origin of the Animals

The wild boars originated from five provinces in the southern half of Sweden. Samples from most of the animals, 92 out of 139 (66%), were obtained from the province of Närke (Table S2). There was no difference between the presence of anti-HEV antibodies, HEV-RNA, or both for animals with different geographical origin (Table S2). In total, there were 21 (16%) wild boars having indications of ongoing or past HEV infection. Ten had ongoing infection based on HEV RNA detection; another 10 had markers for past infection (anti-HEV antibodies), and one additional animal had both detectable HEV RNA and anti-HEV antibodies (Table 2). There was no significant difference between the age of the animal and the prevalence of markers for past or current HEV infection. 

The 69 moose originated from six provinces, from Västerbotten in the north to Västergötland in the south (Table S3). Anti-HEV antibodies, HEV RNA, or both were found in 19 animals from four provinces (Västerbotten, Jämtland, Västmanland, and Västergötland). These HEV markers for past or ongoing infections were significantly more prevalent in moose than in wild boar (19 out of 69 versus 21 out of 139; *p* = 0.04 Fisher’s exact test). In all, 12 moose had detectable HEV RNA; in five of them HEV RNA could be detected in both serum and fecal materials. Two moose had both detectable anti-HEV antibodies and HEV RNA. One was older and one was younger than one year. The prevalence of HEV markers did not differ between moose younger or older than one year. 

All deer originated from three provinces in the southwestern part of Sweden. The animals with anti-HEV antibodies originated from the provinces of Västmanland and Västergötland (Table S4). Since all 97 available sera and stool samples from roe deer, fallow deer, and red deer did not test positive in the PCR assays used, the prevalence of HEV markers could only be based on detectable anti-HEV antibodies, which was 6.9% (two out of 29) in roe deer, and 7% (one out of 14) in red deer. None of the five fallow deer had detectable anti-HEV antibodies.

### 3.4. Phylogenetic Analysis 

**Figure 1:**
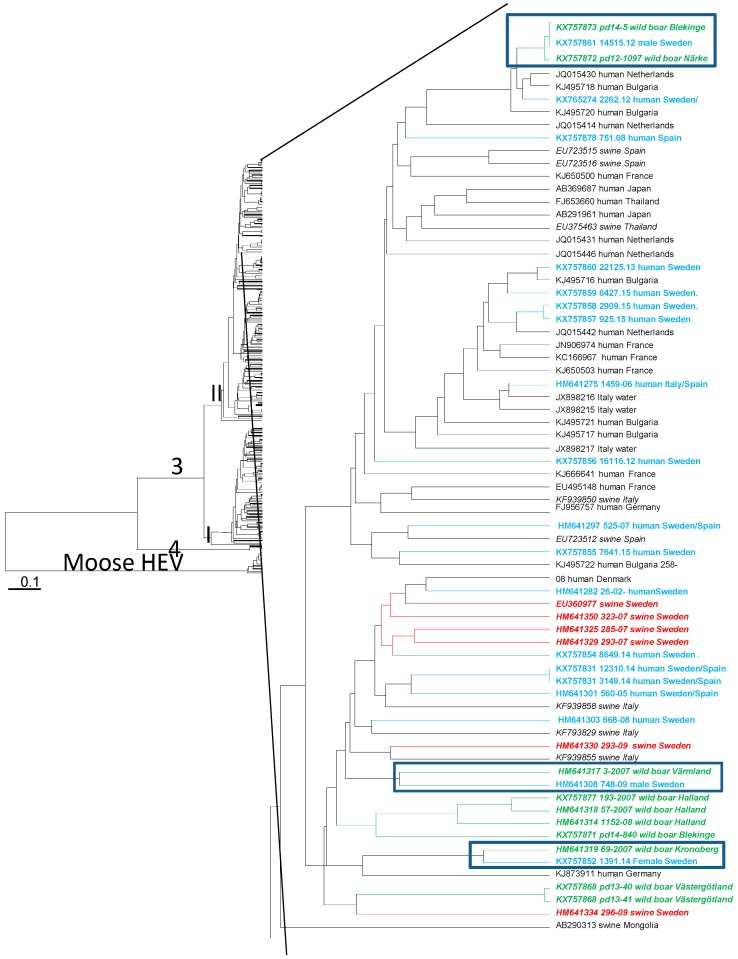
Unweight pair-group method using arithmetic averages (UPGMA) phylogenetic tree formed by 335 nucleotides of partial open reading frame 1 (ORF1) of 361 HEV sequences representing HEV3, HEV4, and moose HEV from this study and obtained from GenBank. The genotypes of the strains are indicated on the branches of the tree. The branch formed by HEV moose strains is indicated. A branch within the 3II clade [37] is enlarged. Accession number or strain designation of the strains in this study together with origin and host of the strains are given at the nodes. The three clades formed by strains from endemic human cases and strains from Swedish wild boar are boxed. Strains from humans infected in Sweden are colored blue, strains from Swedish pigs are colored red, and strains from Swedish wild boar are colored green.

Strains from six wild boars could be amplified and sequenced in the ORF1 region. Phylogenetic analyses showed that all wild boar HEV strains and the 14 strains from human endemic cases were of genotype 3 and were found in the clade previously designated 3II in the phylogenetic tree based on HEV3 strains (Figure 1) [37]. Wild boar strains from animals killed by the same hunter team were similar to each other, although no geographical clades formed by the strains were apparent. Three humans were infected by strains similar to wild boar HEV strains. The strain isolated from one patient from the province of Södermanland, who often consumed wild game meat, was identical to a strain from a wild boar from the province of Blekinge in Southern Sweden. This strain was also similar to a wild boar HEV strain from Närke in Central Sweden (Figure 1). A hunter from the province of Värmland was infected by a strain related to a wild boar from the same province, and a female was infected by a strain similar to a wild boar strain from Småland (Figure 1). HEV strains from wild boar from the provinces of Halland and Blekinge formed one clade. The wild boar strains from Västergötland were distantly related to strains from Swedish pigs in Närke (Figure 1). 

Phylogenetic analysis of six moose strains in ORF1 confirmed the separate branch formed by these strains (Figure 1). No geographical clades were formed by the strains, although most sequenced moose HEV strains originated from the province Västergötland. None of the human or wild boar HEV strains was found to be similar to moose HEV. 

## 4. Discussion

This study shows that approximately 10% of wild game that are hunted for human consumption has ongoing hepatitis E virus infection, and a further 7% have markers of previous infection. Regional differences in the prevalence of HEV markers were not observed. The sampling was, however, uneven, with most wild boars being sampled from the province of Närke, most moose samples from Södermanland, Västmanland, and Västergötland, and the deer samples from Västergötland and Västmanland. Therefore, regional differences in prevalence and spread of specific strains, as previously assumed [38], could not be evaluated in this study. However, regional differences in HEV prevalence, between 5% and 88%, related to animal density, have been shown for wild boar in Poland, Germany, and Italy [39,40,41,42,43]. 

The frequency of HEV-RNA-positive wild boar in this study, which showed that 4% of the animals excreted HEV in their feces, confirmed our previous findings on samples taken between 2005 and 2007, which showed that 4.8% of the wild boars that were older than one year excreted HEV [38]. This finding indicates that the prevalence of HEV infection in Swedish wild boars has been stable for almost ten years. There was, however, no significant difference in prevalence between the younger and older animals with anti-HEV, as it might have been expected if the infection had been circulating in the wild boar population for a longer time. The reason for this may be either that the assay used did not detect low-level wild boar anti-HEV antibodies, or that the animals do not have life-long protection against HEV and may have become re-infected after loss of their immunity. This may also explain why there were as many older as younger animals secreting HEV in their stool. The high prevalence of HEV in wild boars, and the equal frequency of animals with anti-HEV antibodies and HEV RNA in serum or feces, has also been shown in studies from other European countries [42,44,45], making this species a plausible reservoir for HEV in many countries. 

The addition of samples from 12 moose confirmed our previous finding that HEV is at least as common as or even more common in moose than in wild boars [34]. HEV was secreted in feces slightly more often by younger than by older animals, but was found in blood at equal frequency regardless of the age of the animal. The present Swedish populations of both moose and wild boar have both their origin in a few animals that spread in the country, since both species were hunted to extinction or almost to extinction before they could establish new populations in the country. Despite this, the genetic variability of the HEV populations is completely different in these species. All of the moose were infected with HEV strains that were genetically very similar to each other, despite the fact that the animals have a large geographical distance from each other. In contrast, the wild boars were infected with a large variety of strains of the virus, even if they were not geographically far from each other. This may indicate that moose is the only host for the moose HEV, which may explain the genetic stability of this virus. On the other hand, the genetically different HEV strains that infect wild boar can also infect domestic swine and perhaps even deer, and thus have a zoonotic spread that can explain the genetic variability of the virus. So far we could not identify a genotype 3 strain in moose or deer, nor have we found a human, wild boar, or deer infected by moose HEV. Further studies are needed to investigate if moose HEV can infect humans directly, or indirectly by infecting wild boar or deer and evolving genetically within these hosts and acquiring the ability to infect humans. In addition, the common assays used for HEV RNA detection in humans may not detect moose HEV RNA. There may thus be humans infected by moose HEV that escaped detection and identification. 

The seroprevalence of HEV was as high for the different cervid species as it was for wild boar. However, HEV could not be identified by PCR in serum or fecal materials from any deer species apart from moose. The reason for the absence of detectable HEV in blood and stool may either be due to a low prevalence or a low number of animals of each investigated species. They may also be infected by a divergent HEV type that could not be detected by the PCR assays used in this study. Absence of HEV RNA in different species of deer has also been reported in Canada [46]. Presence of HEV RNA in samples from red deer and roe deer was difficult to confirm by sequencing in a study from the Czech Republic [47], and anti-HEV antibodies were not found in any red deer analyzed in Poland or in any roe deer analyzed in Germany [39,48]. However, HEV RNA has frequently been found in samples from red deer in Spain and Italy, and the strains infecting these animals were shown to be of genotype 3, similar to strains infecting wild boars and swine in these countries [49,50]. This indicates that these animals may also be potential sources for HEV transmission in Sweden, since they had detectable anti-HEV antibodies, even if no HEV RNA could be detected in samples from the studied animals. 

It has been shown that hepatitis E can be transmitted by the consumption of undercooked or raw food products produced from infected wild boars and deer in Japan and Korea [51,52,53]. In Japan, the wild boar and sika deer were infected with HEV3, and in South Korea a person having ingested meat from a roe deer was infected with HEV4 [53], indicating that different deer species may be infected with different genotypes. If European roe deer, wild boar, or both were infected with HEV4 strains, this may explain the low number of human infected by HEV4 amoug Europeans without history of travel before the onset of hepatitis E [11,54,55]. 

The zoonotic origin and spread potential of HEV is thus certain and has been clearly demonstrated [56]. Despite this, the route of transmission for the human endemic cases is usually difficult to determine. People with an active outdoor life in the woods may encounter fecal materials or other remains from HEV-infected animals that may cause zoonotic infection. The route of infection may in those cases be difficult to confirm if the isolated HEV strains are not sequenced and characterized. In this study, we found at least three humans infected with HEV strains that were similar or identical to strains infecting wild boar. The probable routes of infection for two of these people were either by direct contact with an infected animal, consumption of wild meat, or both. However, there must be alternative routes of infection than ingestion of infected wild game or pork meat, since some of the people infected are vegetarians or have not consumed meat before falling ill with hepatitis E. The high seroprevalence of 17% of the Swedish population having encountered HEV [27,28,29], and the relatively high frequency of Swedish blood donors with HEV RNA in their plasma [57] also stress that there must be as yet undiscovered transmission routes. One route may be through the irrigation of vegetables or berries with HEV-contaminated water, as it has been shown for berries [58]. In this study, one probable route of infection identified for a female infected with a wild boar HEV strain was her frequent consumption of blue berries and raspberries, which may have been contaminated. Urine or fecal materials from infected animals may contaminate water or berries and mushrooms, since many of the infected animals surveyed in this study secreted HEV in feces. It is known that at least pigs are secreting HEV in urine [59], and HEV RNA has been found in moose kidney [17]. It is not known for how long HEV remains infectious outside its hosts, and this needs to be studied further. For each endemic case, there is thus a need for a thorough investigation by a public health officer. Furthermore, it is important to characterize the infecting strains from each case by phylogenetic analysis in order to identify possible unknown routes of HEV transmission. 

## Figures and Tables

**Table 1:** Primers and probes used in the real-time RT-qPCR assays for detection of HEV1-HEV4, and moose-HEV.

Primer/Probe Designation	Primer Sequence from 5′ to 3′	Position	Reference
HEV genotype 1–4			
HEVF	CGGTGGTTTCTGGGGTGAC	5292–5310 ^1^	This study
HEVR	GAAGGGGTTGGTTGGATGAA	5364–5345 ^1^	This study
HEVP	CGGGTTGATTCTCAGCCCTTCGC FAM	5311–5333 ^1^	This study
Moose-HEV			
HEVF8	AGGTGGTGGTTGGGGCCCT	5037–5055 ^2^	[34]
HEVR8	TGGCGAATGGGTTTGAGGGG	5113–5094 ^2^	[34]
HEVP8	CGCCTCGACTCGCAGCCATTTGC FAM	5056–5078 ^2^	[34]

^1^ Hepatitis E virus isolate swX07-E1, EU360977; ^2^ Hepatitis E virus isolate AlgSwe2012, KF951328.

**Table 2:** Hepatitis E markers identified in serum and fecal samples from the different species of animals.

Animal Species	No. Animals	No. of Anti-HEV Positive Sera/Number Tested (%)	No. of HEV RNA Positive Serum Samples/Number Tested (%)	No. of HEV RNA Positive Stool Samples/Number Tested (%)	No. of Animals with Any HEV Marker (%)
Wild boar *Sus scrofa*	139	11/134 (8.2)	7/134 (5.2)	5/130 (3.8)	21 ^1^ (16)
Moose *Alces* *alces*	69	9/66 (14)	10 ^2^/66 (15)	7 ^2^/63 (11)	19 ^3^ (28)
Roe deer *Capreolus capreolus*	30	2/29 (7)	0/29	0/27	2
Red deer *Cervus* *elaphus*	15	1/14	0/14	0/13	1
Fallow deer *Dama* *dama*	7	0/7	0/7	0/7	0
**TOTAL**	260	23/250 (9.2)	17/250 (6.8)	12/240 (5)	43/260 (17)

^1^ One wild boar had HEV RNA both in blood and in fecal materials; ^2^ There were five moose with HEV RNA in both their serum and fecal materials; ^3^ Two moose had both detectable anti-HEV antibodies and HEV RNA.

## Supplementary Materials

The following are available online at www.mdpi.com/1999-4915/8/9/259/s1: Table S1: Primers used for PCR amplification of fragments for sequencing, Table S2: Frequency of hepatitis E virus markers in sera and fecal samples from wild board (*Sus scrofa*) in relation to county of origin, Table S3: Frequency of hepatitis E virus markers in sera and fecal samples from moose (*Alces alces*) in relation to county of origin, Table S4: Frequency of hepatitis E virus markers in sera and fecal samples from roe deer (*Capreolus capreolus*), fallow deer (*Dama dama*) and red deer (*Cervus elaphus*) in relation to county of origin.
