# Supplementary material for: Markers for Ongoing or Previous Hepatitis E Virus Infection Are as Common in Wild Ungulates as in Humans in Sweden
Source: Viruses. 2016 Sep 19;8(9):259. doi: 10.3390/v8090259 (PMC5035973; doi:10.3390/v8090259)
Supplement: Supplementary file 1 [file viruses-08-00259-s001.pdf]

Supplemental material

Table S1. Primers used for PCR amplification of fragments for sequencing

Primer designation	Primer sequence from 5´to 3´	Position [1]
ISP-4232 0	GTATTTCGGCCTGGAGTAAGAC	4267-4288
ISP-4232 A	GCATTTCRGCYTGGAGYAGAC	
ISP-4232 B	GTATATCGGCCTGGAGYAAGAC	
ISP-4232 C	GTATCTCRGCYTGGAGYAAAAC	
ISP-4232 D	GTATCTTCCGCTGGAGTAAGAC	
ISP-4232 E	GYATATCGGCTTGGAGCAAGAC	
ISP-4232 F	GTATATCWGCCTGGYCCAAAAC	
ISP-4232 G	GYATTTCAGCGTGGTCAAAGAC	
ISP-4232 H	GCATTTCGGCGTGGTCAAAAAC	
EAP-4576 0	AGGGTGCCGGGCTCGCCGGA	4630-4611
EAP-4576 A	AGGGTRCCAGGCTCACCAGA	
EAP-4576 B	AGGGTGCCRGGYTCRCCGGA	
EAP-4576 C	AGTGTGCCDGGCTCGCCAGA	
EAP-4576 D	AAGCTGCCCGGCTCACCAGA	
EAP-4576 E	AGYGTRCCRGGCTCACCRGA	
EAP-4576F	AAYGTACCGGGCTCACCRGA	
EAP-4576G	AAYGTGCCRGGCTCACCRGA	
EAP-4576H	AGAGTCCCCGGCTCTCCAGA	
IAP-4561O	TCACCGGAGTGYTTCTTCCAGAA	4618-4596
IAP-4561A	TCACCRGAGTGYTTCTTCCAGAA	
IAP-4561B	TCACCAGARTGCTTCTTCCAGAA	
IAP-4561C	TCACCRGARTGCTTCTTCCAAAA	
IAP-4561D	TCACCKGAATGYTTCTTCCATCG	
IAP-4561E	TCACCAGARTGTTTYTTCCATCG	
IAP-4561F	TCTCCAGAGTGCTTTTTCCAGCA	
IAP-4561G	TCGCCRGAGTGCTTCTTCCARAA	
IAP-4561moose	TCACCAGAATGTTTTTTCCAACTG	

[1]Hepatitis E virus isolate swX07_E1_ *EU360977*

Table S2. Frequency of hepatitis E virus markers in sera and fecal samples from wild boar (*Sus scrofa*) in relation to county of origin.

County of origin	No. of animals	Anti-HEV no. of positive/total samples analysed (%)	No HEV RNA pos animals (%)	No. of wild boars with any HEVmarker (%)
Blekinge	8	2	2	3
Närke	92	4/88	6	10
Södermanland	3	1	0	1
Västergötland	22	2/21	2	4
Västmanland	12	2	1	3
Unknown region	2	0	0	0
Total	139	11/134 (8.2)	11 (7.9)	21 (15)

Table S3. Frequency of hepatitis E virus markers in sera and fecal samples from moose *(Alces alces)* in relation to county of origin.

County of origin	No. of animals	No. of anti-HEV positive/total no. samples analysed (%)	No HEV RNA pos animals (%)	No. of moose with any HEVmarker (%)
Södermanland	9	0/9	0	0
Västergötland	16	4/14	4	6[1]
Västmanland	15	3/15	4	7
Värmland	1	0/1	0	0
Jämtland	15	1/15	3	4
Västerbotten	10	0/10	1	1
Unknown region	3	1/3	0	1
Total	69	9/67 (13)	12 (17)	19[1] (28)

[1]Two moose had both detectable anti-HEV and HEV RNA

Table S4. Frequency of hepatitis E virus markers in sera and fecal samples from roe deer *(Capreolus capreolus*), fallow deer (*Dama dama*) and red deer*(Cervus elaphus)* in relation to county of origin.

Deer species/ County of origin	No. of animals	No. of anti-HEV positive/total samples analysed (%)	No HEV RNA pos animals (%)	No. of deer with any HEV marker (%)
Roe deer				
Södermanland	1	0/1	0	0
Västergötland	15	1/14	0	1
Västmanland	11	1/11	0	1
Värmland	2	0/2	0	0
unknown	1	0/1	0	0
Subtotal	30	2/29 (6.9)	0	2 (6.7)
Fallow deer				
Södermanland	1	0/1	0	0
Västergötland	2	0/2	0	0
Västmanland	4	0/4	0	0
Subtotal	7	0/7	0	0
Red deer				
Västmanland	15	1/14	0	1
Total	52	3/57 (6)	0	3 (5.8)
